# Emergency Physician-Performed Bedside Ultrasound of Gastric Volvulus

**DOI:** 10.7759/cureus.9946

**Published:** 2020-08-22

**Authors:** Keyon Shokraneh, Jordan Johnson, Gabriel Cabrera, Eric J Kalivoda

**Affiliations:** 1 Emergency Medicine, Hospital Corporation of America Healthcare West Florida Graduate Medical Education Consortium/University of South Florida Morsani College of Medicine, Brandon Regional Hospital, Brandon, USA

**Keywords:** point-of-care ultrasound, ultrasonography, gastric volvulus, emergency department

## Abstract

Gastric volvulus (GV) is a seldomly encountered life-threatening condition that necessitates rapid diagnosis in the emergency department (ED). The diagnosis of GV is traditionally established with cross-sectional imaging and/or endoscopy with surgical confirmation. The potential role of point-of-care ultrasound (POCUS) as a bedside tool to support the early identification of GV by emergency physicians (EPs) has not been thoroughly investigated. This case report describes the expeditious diagnosis and ED management of acute GV by implementing EP-performed POCUS into critical decision making.

## Introduction

Gastric volvulus (GV) is a rare surgical emergency with mortality rates approaching 30%-50% when complications occur [1-4]. The pathophysiology of GV is classified by an abnormal rotation of the stomach along the longitudinal (organoaxial) axis, transverse (mesenteroaxial) axis, or a combined axis. Gastric strangulation and complications of necrosis, perforation, and/or septic shock can result when rotation exceeds 180° with complete closed-loop obstruction. Primary GV is usually attributed to neoplasm, adhesions, or abnormal stomach attachments, whereas paraesophageal hernia is the most common etiology of secondary GV in adults [2,4].

Timely recognition and diagnosis of GV is imperative to avoid detrimental delays in the emergency department (ED) intervention and definitive management. Plain radiography and CT are the initial diagnostic modalities typically employed when GV is suspected; however, the utilization of ultrasonography to support the diagnosis has not been systematically studied [5-11]. The use of point-of-care ultrasound (POCUS) to diagnose acute abdominal emergencies, such as small bowel obstruction, is within the scope of practice for emergency physicians (EPs) [12,13]. Similarly, the identification of acute gastric outlet obstruction (GOO) (secondary to underlying malignancies and peptic ulcer disease) with POCUS has been described in two previous case reports [14,15]. To the best of our knowledge, this is the first case report describing EP-performed POCUS of GV secondary to paraesophageal hernia.

## Case presentation

A 69-year-old male with a past medical history of hypertension, atrial fibrillation, antineutrophil cytoplasmic antibody (ANCA) vasculitis, chronic kidney disease, gastroesophageal reflux disease, and hiatal hernia presented to the ED with chief complaint of abdominal pain. He described a one-day history of gradual-onset, constant, dull, moderate severity, non-radiating epigastric discomfort with associated abdominal distention, nausea, and decreased appetite. He reported two episodes of vomiting prior to arrival. He denied fevers, chills, diarrhea, constipation, dark or bloody stools, flank pain, back pain, urinary complaints, chest pain, shortness of breath, palpitations, or syncope. Initial vital signs were temperature 36.8°C, blood pressure 157/106 mmHg, heart rate 83 beats per minute, respiratory rate 14 breaths per minute, and oxygen saturation of 95% on room air. Physical examination revealed an elderly male in moderate distress secondary to pain. Significant tenderness to palpation in the epigastrium and left upper quadrant was elicited on abdominal examination without rebound, guarding, or peritoneal signs. The remainder of the physical examination was unremarkable. Electrocardiogram (ECG) revealed normal sinus rhythm without acute ischemic changes.

**Figure 1:**
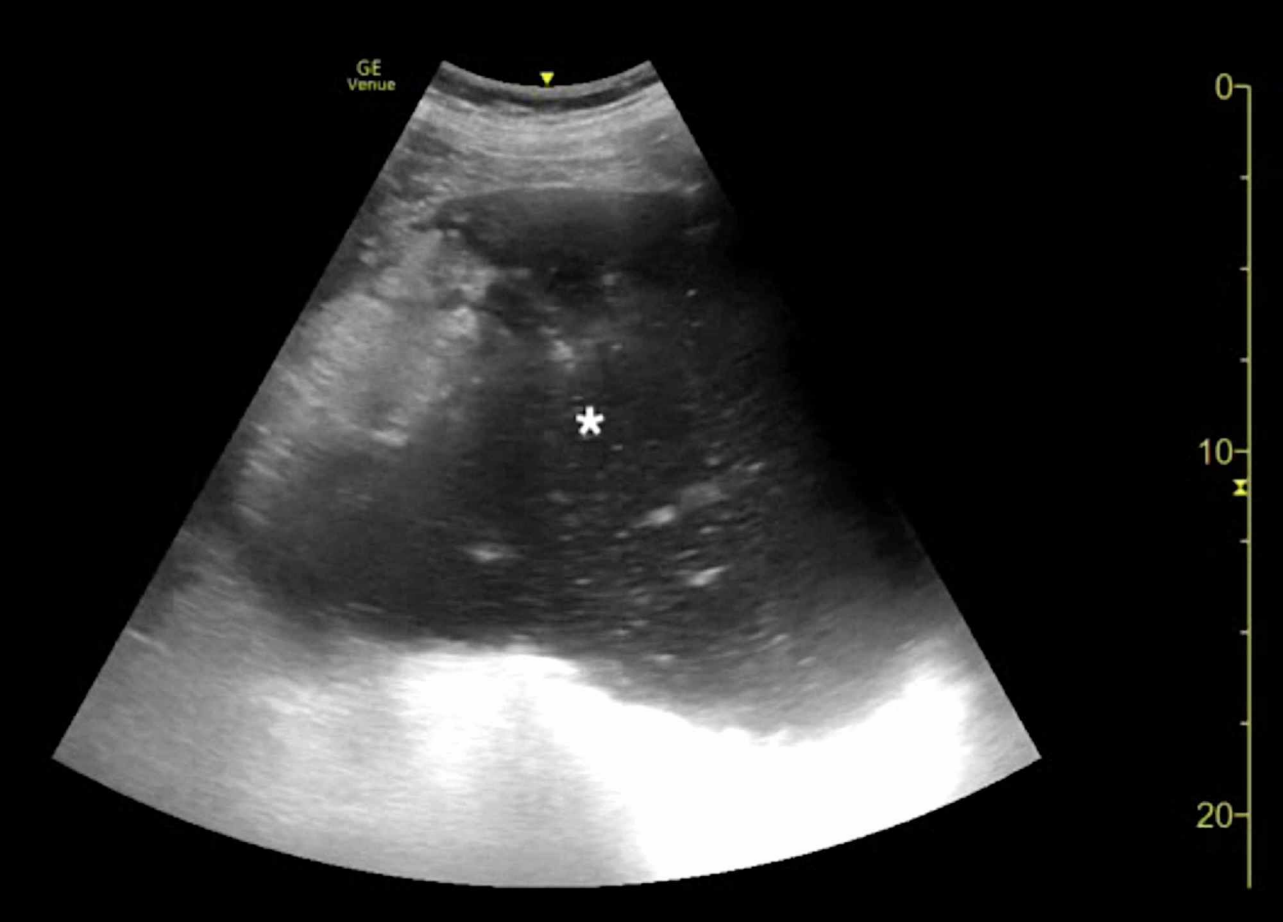
Point-of-care ultrasound of gastric volvulus demonstrating a distended, fluid-filled stomach (asterisk).

**Figure 2:**
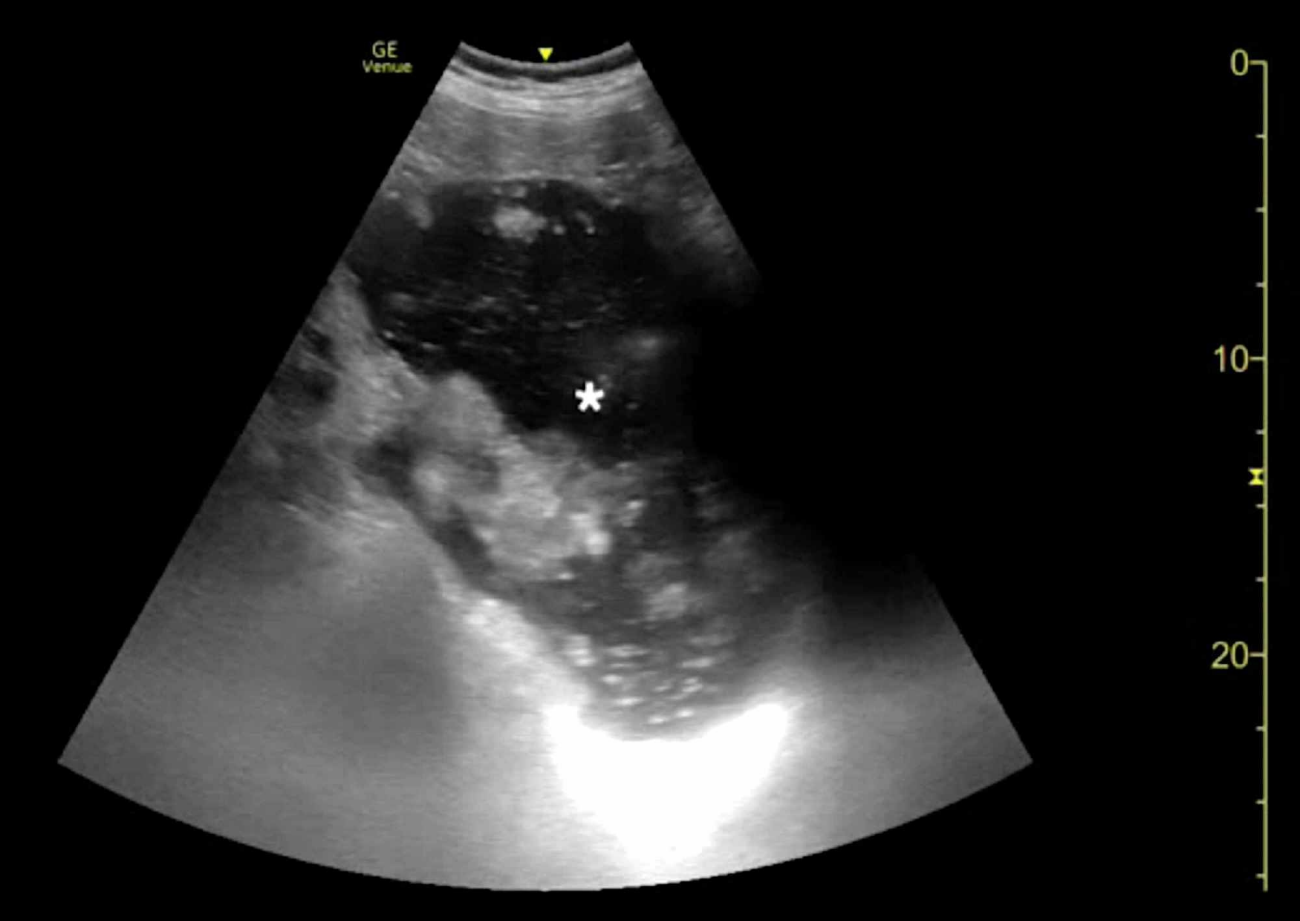
Point-of-care ultrasound of gastric volvulus demonstrating a distended, fluid-filled stomach (asterisk).

The clinical presentation was concerning for an acute intra-abdominal emergency. CT of the abdomen and pelvis, laboratory studies, pain medications, antiemetics, and intravenous (IV) fluids were ordered. Emergency medicine resident physicians and an ultrasound fellowship-trained attending EP then performed an abdominal POCUS examination. POCUS was notable for a large, dilated, fluid-filled stomach highly concerning for an obstructive process (Figures 1, 2; Videos 1, 2).

**Video 1:** Point-of-care ultrasound of gastric volvulus demonstrating a distended, fluid-filled stomach.

**Video 2:** Point-of-care ultrasound of gastric volvulus demonstrating a distended, fluid-filled stomach.

**Figure 3:**
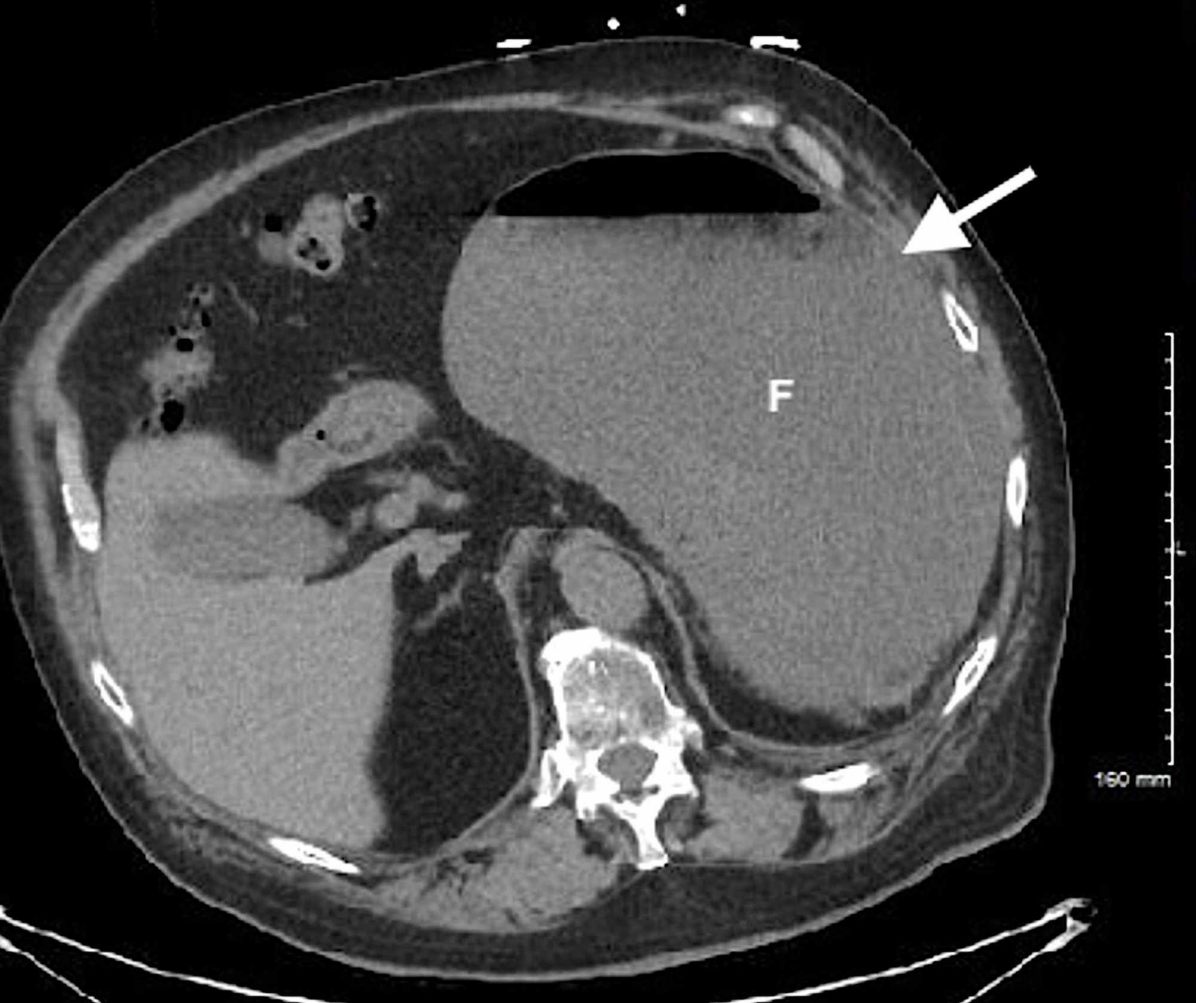
CT of the abdomen/pelvis (axial view) demonstrating gastric volvulus with a fluid-filled distended stomach (solid arrow). F, gastric fundus.

**Figure 4:**
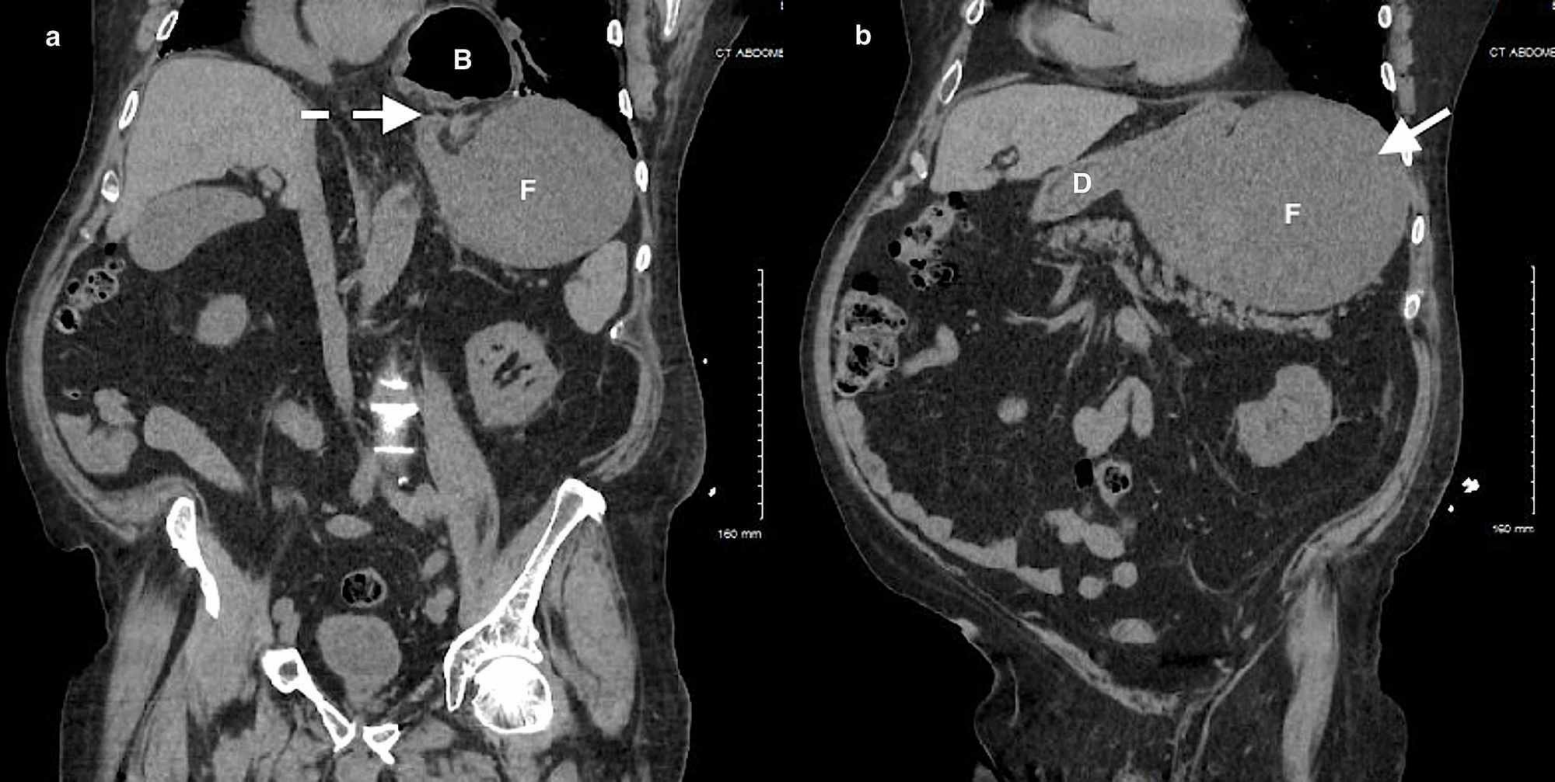
CT of the abdomen/pelvis (coronal views) demonstrating gastric volvulus with (a) malrotation (dotted arrow) and a (b) fluid-filled distended stomach (solid arrow). B, gastric body; F, gastric fundus; D, duodenum.

**Figure 5:**
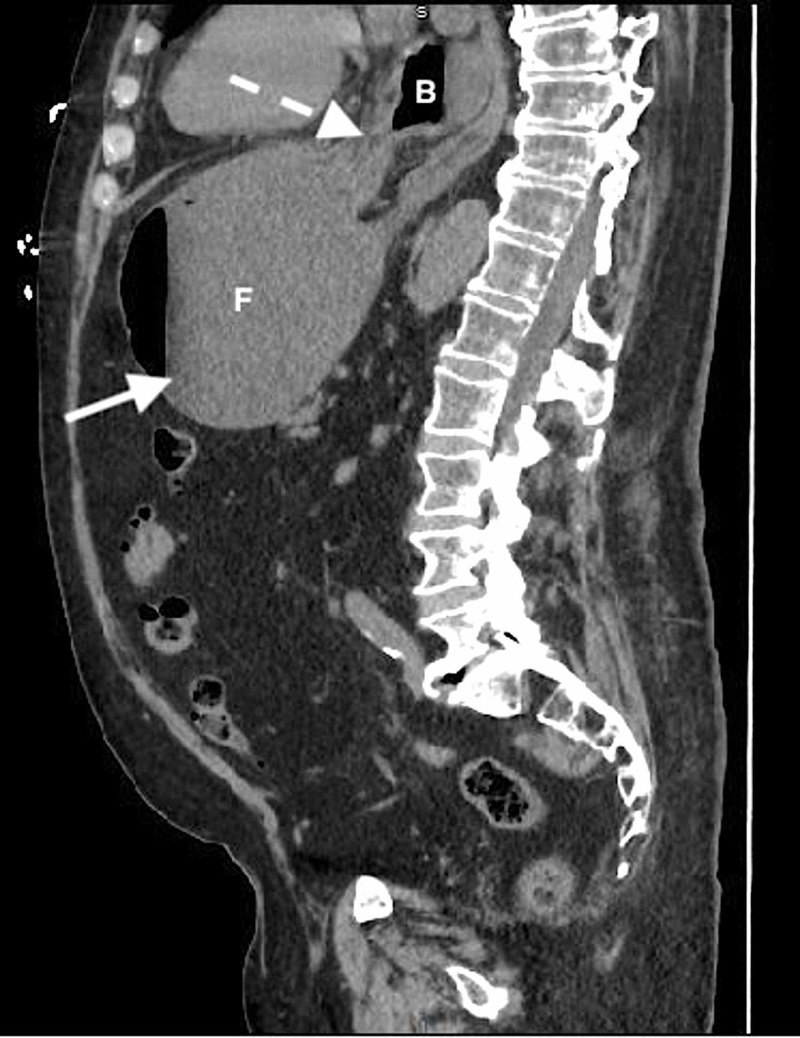
CT of the abdomen/pelvis (sagittal view) demonstrating gastric volvulus with a fluid-filled distended stomach (solid arrow) and malrotation (dotted arrow). B, gastric body; F, gastric fundus.

**Figure 6:**
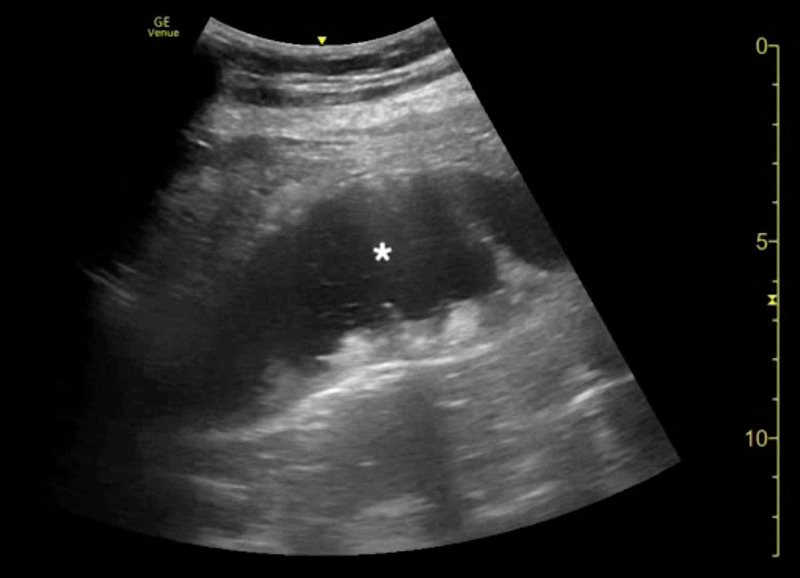
Point-of-care ultrasound of gastric volvulus post-nasogastric tube insertion demonstrating stomach decompression (asterisk).

CT imaging was subsequently expedited based upon the POCUS findings. CT of the abdomen/pelvis revealed a moderate to large hiatal hernia and a distended edematous stomach with partial obstruction due to GV (Figures 3-5). General surgery was consulted emergently and evaluated the patient at bedside in the ED. Multiple prolonged attempts at nasogastric tube (NGT) insertion were initially unsuccessful. NGT placement was ultimately successful yielding ~2,000 mL of non-bilious stomach contents, and repeat POCUS confirmed gastric decompression (Figure 6). Laboratory analysis was unremarkable for acute clinically significant abnormalities.

The patient was admitted for further management of GV. Gastroenterology was consulted for emergent esophagogastroduodenoscopy (EGD) that confirmed the presence of a partial organoaxial volvulus without evidence of gastric ischemia. EGD did not reveal any underlying pathology in the upper gastrointestinal tract with the exception of large hiatal hernia and mild gastritis. General surgery then performed laparoscopic reduction of GV, hiatal hernia repair with mesh, and Nissen fundoplication. The patient was soon tolerating a full liquid diet and closely managed post-operatively by general surgery and gastroenterology. The patient was discharged home in stable condition on hospital day 6 with instructions for outpatient surgery follow-up.

## Discussion

The ED evaluation of acute abdominal pain requires a diligent and meticulous approach to identify critical underlying pathology. Symptoms may be vague or non-specific, and the classic constellation of clinical findings may not be present. Acute GV with complete obstruction will oftentimes be recognized with the classical Borchardt’s triad of intractable retching, severe epigastric abdominal pain and distention, and difficulty or inability to pass an NGT [2,4]. Importantly, GV can mimic other common causes of epigastric pain, such as gastroesophageal reflux disease or peptic ulcer disease, and may even present with complaint of chest pain [16,17]. Our case patient notably did report epigastric pain, but did not experience any further intractable retching or vomiting during his ED course; we did experience significant difficulty with NGT insertion before it was eventually placed, suggesting an incomplete obstructive process. GV can be a challenging diagnosis in the ED setting; therefore, EPs should maintain a higher degree of clinical suspicion in patients with a known underlying hiatal hernia.

The choice of initial imaging study is significant when there is suspicion for GV, as diagnostic delay can lead to fatal complications. CT imaging is highly reliable and accurate, with two findings (a normal gastropyloric transition zone and the antrum in an abnormal location) with 100% sensitivity and specificity for the diagnosis of acute GV [18,19]. One retrospective study investigated radiologists’ accuracy of CT interpretation for GV, with reported 90% overall accuracy [19]. Interestingly, radiologists were able to make the critical distinction between GV and large hiatal hernia, which are notoriously difficult entities to distinguish, and especially notable because most GV cases are associated with paraesophageal hernias.

POCUS is an effective non-invasive imaging modality for the bedside assessment of a multitude of abdominal emergencies. Moreover, POCUS offers the vital advantages of avoiding radiation exposure and is readily available for repeat serial examinations as dictated by the clinical scenario. Previous literature has reported the utilization of POCUS for identifying gastric dilation in cases of GOO [14,15]. We discerned comparable sonographic findings in our case, with visualization of a severely dilated stomach and layering of gastric contents similar to the “black-and-white cookie” sign of GOO [15]. We were also able to use POCUS to dynamically observe successful gastric decompression. EPs should consider utilizing bedside ultrasonography for the rapid verification of appropriate NGT placement. This case highlights another promising application of POCUS as an initial imaging modality to support an earlier ED diagnosis of GV.

## Conclusions

GV is a life-threatening surgical emergency that warrants prompt EP recognition and management. Implementation of POCUS findings into clinical decision making has the potential to facilitate an expedited diagnosis. Further larger-scale studies should investigate the diagnostic accuracy and ideal role of EP-performed POCUS as a first-line imaging strategy for GV.
